# Acute stress leaves fear generalization in healthy individuals intact

**DOI:** 10.3758/s13415-021-00874-0

**Published:** 2021-02-24

**Authors:** Franziska Magdalena Kausche, Gundula Zerbes, Lea Kampermann, Jana Christina Müller, Klaus Wiedemann, Christian Büchel, Lars Schwabe

**Affiliations:** 1 Department of Cognitive Psychology, Universität Hamburg, Von-Melle-Park 5, 20146 Hamburg, Germany; 2 Institute for Systems Neuroscience, University Medical Center Hamburg-Eppendorf, 20246 Hamburg, Germany; 3 Department of Psychiatry and Psychotherapy, University Medical Center Hamburg-Eppendorf, 20246 Hamburg, Germany

**Keywords:** Stress, Fear generalization

## Abstract

Because threatening situations often occur in a similar manner, the generalization of fear to similar situations is adaptive and can avoid harm to the organism. However, the overgeneralization of fear to harmless stimuli is maladaptive and assumed to contribute to anxiety disorders. Thus, elucidating factors that may modulate fear (over)generalization is important. Based on the known effects of acute stress on learning, which are at least partly due to noradrenergic arousal, we investigated whether stress may promote fear overgeneralization and whether we could counteract this effect by reducing noradrenergic arousal. In a placebo-controlled, double-blind, between-subjects design, 120 healthy participants underwent a fear-conditioning procedure on Day 1. Approximately 24 hours later, participants received orally either a placebo or the beta-adrenergic receptor antagonist propranolol and were exposed to a stress or control manipulation before they completed a test of fear generalization. Skin conductance responses as well as explicit rating data showed a successful acquisition of conditioned fear on Day 1 and a pronounced fear generalization 24 hours later. Although physiological data confirmed the successful stress manipulation and reduction of noradrenergic arousal, the extent of fear generalization remained unaffected by stress and propranolol. The absence of a stress effect on fear generalization was confirmed by a second study and a Bayesian analysis across both data sets. Our findings suggest that acute stress leaves fear generalization processes intact, at least in a sample of healthy, young individuals.

## Introduction

The experience of a threatening stimulus automatically triggers the subjective experience of fear, an adaptive emotion that helps us to avoid future harm. Because most stimuli do not occur in the exact same manner across situations, the ability to generalize fear to stimuli resembling an initial threat stimulus is highly adaptive. This fear generalization is reflected in a fear gradient, in which the fear response is highest towards the original threatening stimulus but spreads, at least in part, to similar stimuli and the lowest response is shown to the most dissimilar stimulus (Lissek et al., 2008; Lissek, Bradford, et al., 2014; Shepard, 1987). Although fear generalization may be generally adaptive, the inability to distinguish threat from safety and the overgeneralization of fear to safe stimuli are maladaptive. In particular, fear overgeneralization is thought to underlie the behavioral symptoms of fear-related disorders or posttraumatic stress disorder (PTSD; Dunsmoor & Paz, 2015; Kaczkurkin et al., 2017; Lis et al., 2020; Lissek, 2012; Lopresto, Schipper, & Homberg, 2016; Morey et al., 2020). Given that anxiety disorders are among the most common mental disorders (Kessler et al., 2005; Wittchen & Jacobi, 2005), identifying factors that may promote the overgeneralization of fear is important. Accordingly, previous studies showed that the extent of fear generalization can be influenced by the intensity of threat (Dunsmoor, Kroes, Braren, & Phelps, 2017), verbal instructions (Vervliet, Kindt, Vansteenwegen, & Hermans, 2010), the degree of anxious personality (Sep, Steenmeijer, & Kennis, 2019), and stimulus similarity and perception (Struyf, Zaman, Hermans, & Vervliet, 2017; Struyf, Zaman, Vervliet, & Van Diest, 2015; Zaman, Ceulemans, Hermans, & Beckers, 2019; Zaman, Struyf, Ceulemans, Beckers, & Vervliet, 2019). On a neural level, findings suggest that adaptive fear generalization requires an intricate balance of excitatory and inhibitory mechanisms of different brain regions. Specifically, studies repeatedly associated activation in the anterior insula (aI), the dorsomedial prefrontal cortex, and the bilateral inferior parietal lobe with fear excitation, whereas activation of the bilateral ventral hippocampus, the ventromedial prefrontal cortex (vmPFC) and the precuneus cortex is associated with fear inhibition (Dunsmoor, Prince, Murty, Kragel, & LaBar, 2011; Greenberg, Carlson, Cha, Hajcak, & Mujica-Parodi, 2013; Lissek, Bradford, et al., 2014; Onat & Büchel, 2015). In line with behavioral studies that showed that fear generalization can be independent of perception (Bennett, Vervoort, Boddez, Hermans, & Baeyens, 2015; Dunsmoor, Martin, & LaBar, 2012; Dunsmoor & Murphy, 2014), studies investigating the neural mechanisms showed partly sharpened fear generalization on a neural level compared to the behavioral level, indicating that processes other than perception add to fear generalization (Onat & Büchel, 2015; Stegmann, Ahrens, Pauli, Keil, & Wieser, 2020).

Stressful events—known to provoke the release of numerous hormones, neurotransmitters and peptides (Joels & Baram, 2009)—are assumed to be a driving force in fear- and stress-related disorders, such as PTSD (de Quervain, Schwabe, & Roozendaal, 2017; Grillon, Duncko, Covington, Kopperman, & Kling, 2007; Shin & Liberzon, 2010; Yehuda, Giller, Southwick, Lowy, & Mason, 1991). Two of the most prominent stress mediators are glucocorticoids and noradrenaline, both of which are known to be major modulators of learning and memory in general (Diamond, Campbell, Park, Halonen, & Zoladz, 2007; Joels, Fernandez, & Roozendaal, 2011; Quaedflieg & Schwabe, 2018; Roozendaal, Okuda, de Quervain, & McGaugh, 2006; Sandi & Pinelo-Nava, 2007; Schwabe, Joels, Roozendaal, Wolf, & Oitzl, 2012). Moreover, there also is evidence that stress affects fear learning processes (Jackson, Payne, Nadel, & Jacobs, 2006; Merz, Elzinga, & Schwabe, 2016; Simon-Kutscher, Wanke, Hiller, & Schwabe, 2019), presumably also driven by glucocorticoids and noradrenergic arousal (Krugers, Zhou, Joels, & Kindt, 2011; Merz, Hamacher-Dang, Stark, Wolf, & Hermann, 2018). Prefrontal and medial-temporal brain areas critically involved in fear generalization (Greenberg et al., 2013; Lissek, Bradford, et al., 2014; Lopresto et al., 2016; Onat & Büchel, 2015) are known to be particularly sensitive to stress and stress mediators (de Kloet, Joels, & Holsboer, 2005; Krugers, Karst, & Joels, 2012; Roozendaal et al., 2006). Furthermore, initial evidence in humans and animals suggests that stress and stress hormones may induce increased fear generalization (Bender, Otamendi, Calfa, & Molina, 2018; Dunsmoor, Otto, & Phelps, 2017; Kaouane et al., 2012; Kolodziejczyk & Fendt, 2020).

Converging lines of evidence from rodent and human studies indicate that stress effects on learning and memory rely on an interaction of noradrenaline and glucocorticoids (Krugers et al., 2012; Roozendaal & Hermans, 2017; Roozendaal, McEwen, & Chattarji, 2009; Roozendaal et al., 2006; Schwabe, Tegenthoff, Hoffken, & Wolf, 2012) and can therefore be blocked by interfering with noradrenergic (or glucocorticoid) signaling. For instance, it has been shown that the effects of stress or glucocorticoids on memory retrieval can be prevented by a pharmacological reduction of noradrenergic activity through the β-adrenoceptor antagonist propranolol (de Quervain, Aerni, & Roozendaal, 2007; Schwabe et al., 2009). Furthermore, fear learning processes per se are susceptible to the administration of propranolol, resulting for example in a reduced contextual fear conditioning or fear memory reconsolidation (de Quervain et al., 2007; Grillon, Cordova, Morgan, Charney, & Davis, 2004; Kindt, Soeter, & Vervliet, 2009). In addition, it is thought that some symptoms of PTSD rely on a heightened responsiveness of the noradrenergic system, which can be reduced by β-adrenergic blockade (Southwick et al., 1999). However, the role of noradrenergic arousal in putative stress effects on fear generalization is, to the best of our knowledge, unknown.

Therefore, the present study was designed to examine whether stress effects on fear generalization require noradrenergic arousal. To this end, participants underwent a 2-day fear generalization paradigm (Onat & Büchel, 2015), in which fear acquisition took place on experimental Day 1. Twenty-four hours later, participants received either a placebo or propranolol and underwent a stress or control manipulation before they completed the critical test of fear generalization. Furthermore, we included a task to assess participants’ perceptual discrimination ability to rule out that fear generalization is merely due to insufficient perceptual discrimination. While we initially planned to test propranolol effects on stress-induced changes in fear generalization, we did not observe significant stress effects on fear generalization in the first place. We therefore added the data of a second study in which we used the exact same stress protocol and the exact same fear generalization paradigm and run a Bayesian analysis across both data sets in order to test explicitly the evidence in favor of the observed absence of a stress effect on fear generalization.

## Methods and materials

### Study I

#### Participants and experimental design

In Study I, we tested 120 healthy participants (61 women, age: *M* = 25.21 years, *SEM* = 0.35 years). This sample size was based on an a priori power analysis with the software G*Power 3.1 (Faul, Erdfelder, Lang, & Buchner, 2007) for our main hypothesis that acute stress increases fear generalization. The analysis revealed that 119 participants are sufficient to detect a medium-sized effect of f = 0.3 with a power of 0.90. Exclusion criteria were a history of any mental or neurological disorder, current medication intake, and drug or tobacco use. In addition, participants were excluded if they had any contraindications for the intake of the beta blocker propranolol. Women were not tested during their menses and those taking hormonal contraceptives were excluded from participation. All participants provided written, informed consent before taking part in the experiment and received a compensation of 60€ for participation. The study protocol was approved by the ethics committee of the State Chamber of Physicians Hamburg and in accordance with the Declaration of Helsinki.

In a double-blind, placebo-controlled, fully crossed, between-subject design with the factors condition (stress vs. control) and drug (propranolol vs. placebo), participants were pseudo-randomly assigned to one of four experimental groups: control + placebo (C+Plac), control + propranolol (C+Prop), stress + placebo (S+Plac), and stress + propranolol (S+Prop). Because successful fear acquisition is a prerequisite for testing stress or noradrenaline effects on fear generalization, we used the successful (explicit) fear acquisition on experimental Day 1 (i.e., US-expectancy rating CS+>CS-) as a predefined criterion for inclusion in the analysis. We based our criterion on the US-expectancy ratings, rather than the SCRs, because SCRs capture specifically arousal-related processes, which can only partly be used to infer fear learning (Lonsdorf et al., 2019). Based on this criterion, 11 participants had to be excluded, leaving a final sample of 109 participants (57 women; age: *M* = 25.29 years, *SEM* = 0.35 years; C+Plac: n = 28, C+Prop: n = 27, S+Plac: n = 29, S+Prop: n = 25).

#### Fear generalization paradigm

**Figure 1.**
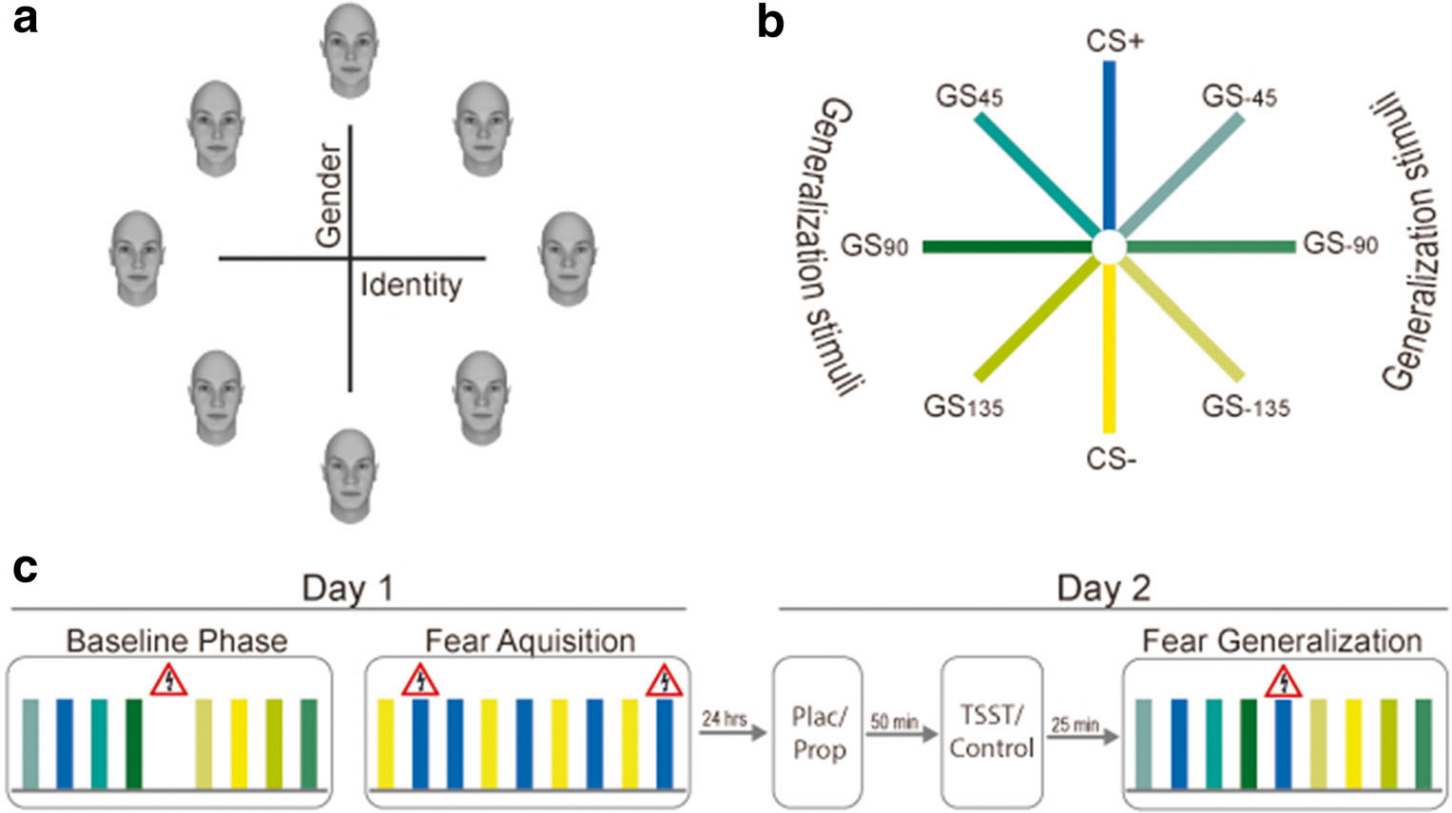
Fear generalization paradigm and stimulus organization. (**A** and **B**) There were eight different face stimuli in total, arranged on a circular similarity continuum with the axes gender and identity. The stimuli in between the CS+ and CS− represent the generalization stimuli (GS). (**C**) Fear generalization paradigm with three phases. On Day 1, the baseline and fear acquisition phases took place. On Day 2, the test of fear generalization followed after the pharmacological and stress/control manipulation. During the baseline phase, the complete set of stimuli (represented by colored bars) was shown to the participants and US were signaled by a shock symbol. During the fear acquisition phase, the two most dissimilar stimuli from opposite sides of the circular similarity continuum were shown to the participants, representing the CS+ and CS−. The CS+ was followed by the US in ~23% of the trials. During the test phase, again the complete set of faces was shown to the participants. To avoid extinction, the CS+ was a reinforced in ~23% of the trials.

To assess fear generalization, we used a recently introduced paradigm (Onat & Büchel, 2015), which included eight face stimuli arranged on a circular similarity continuum along two axes (x-axis: gender; y-axis: identity; Figure 1A). The circular set-up allowed us to investigate a two-sided fear-tuning profile. The opposite points of this circle represent a pair of most dissimilar faces and served as CS+ and CS−, respectively, counterbalanced across participants and groups. In between the CS+ and CS−, stimuli represented the generalization stimuli (GS), which were quantified in their distance to the CS+ (Figure 1B). An unpleasant but not painful electric shock served as unconditioned stimulus (US). Face stimuli were shown for 1.5 sec. During shock trials, the US was presented after 1.4 sec and co-terminated with face offset. The mean intertrial interval was 3.5 sec, ranging between 1.5 and 5.5 sec. To optimally control for participants’ attention to the faces, a fixation cross appeared 1 sec before stimulus onset in the middle of the screen and moved with its onset to the forehead of the face. In the middle of the trial, the cross moved to the chin of the face stimulus and disappeared with stimulus offset.

The paradigm comprised a baseline phase, a fear acquisition phase, and a test of generalization (Figure 1C). The baseline phase served as a control for any a priori differences between the faces. Therefore, during the baseline phase, the complete set of faces was shown to the participants. To maintain a comparable level of arousal due to electrical stimulation across all phases of the paradigm, the phase included 10 shock trials (i.e., the same number of US that was administered as in the fear generalization test phase). Importantly, however, the US was always signaled by a shock symbol to ensure full predictability and prevent any association of the shock with any of the faces. In total, the baseline phase contained 293 trials (each face was shown ~34 times) and lasted for approximately 29 minutes. During the fear acquisition phase, only two faces, i.e., the most dissimilar faces, were presented. In ~23% of the trials, one of the faces (CS+) was followed by the US, whereas the other face (CS−) was never paired with the US. Altogether, 123 trials were presented (duration: ~15 minutes). The reinforcement schedule was based on the study by Onat & Büchel (2015), which showed that this schedule was sufficient for participants to reliably learn the CS-US association. In the fear generalization phase, the complete set of faces was shown again to the participants. In contrast to the baseline phase, however, the US followed the presentation of the CS+ in ~23% of the trials to avoid extinction learning to the CS+. The test phase also contained 293 trials and lasted for approximately 29 minutes.

To ensure attention to the faces, participants were prompted to respond to oddball targets (faces with artificially added freckles, ~10 trials) in every phase. Moreover, after each phase, each face was presented two times in randomized order and US-expectancy ratings were assessed using a visual analog scale (VAS; anchors: “1” = certain, no shock; “10” = certain, shock) to measure explicit fear learning.

At the end of the generalization phase, all of the eight face stimuli were shown to the participants as shown in Figure 1A but in a randomized circular order. Participants had to indicate which of the faces was followed by the shock, i.e., to indicate the CS+ face.

To rule out that potential fear generalization is just based on a failure to perceptually differentiate between the faces, we also assessed participants’ perceptual discrimination ability. To avoid that participants pay too much attention to differences between the faces and thereby diminishing possible effects of fear generalization, we conducted this discrimination task after the fear generalization phase. In this task, two faces were presented successively and participants were asked to rate the faces as being the same or different. In total, the discrimination task consisted of 192 trials.

#### Stress manipulation and control condition

Participants in the stress condition were exposed to the Trier Social Stress Test (TSST; Kirschbaum, Pirke, & Hellhammer, 1993), a standardized protocol for experimental stress-induction in humans that reliably increases subjective stress levels and activates both the autonomic nervous system and the hypothalamus-pituitary-adrenal axis (Kirschbaum et al., 1993). The TSST consists of a mock job interview and a mental-arithmetic task. First, participants had to prepare and deliver a 5-minute free speech, in which they applied for a job tailored to their interests, followed by a challenging 5-minute mental arithmetic task (counting backwards from 2,043 in steps of 17), while being evaluated by a rather cold and nonresponsive panel of two experimenters, both dressed in white lab coats. In addition, participants were videotaped and saw themselves on a screen during task performance. Participants in the control condition gave a 5-minute talk about a topic of their choice, followed by a 5-minute simple arithmetic task (counting forward from zero in steps of 15). Neither an evaluative committee, nor a video camera, were present during the control manipulation.

To validate the successful subjective stress induction, participants rated the difficulty, unpleasantness, and stressfulness of the task on a VAS (anchors: 0 = “not at all”; 100 = “extremely”). In addition, we measured subjective and physiological stress indicators at several time points across the experiment: at the beginning of Day 2, 50 minutes after drug intake (see below), during the TSST or control manipulation (only vital signs), after the TSST/control manipulation (65 minutes after drug intake), before the test of fear generalization (75 minutes after drug intake), after the test of fear generalization (105 minutes after drug intake) and at the end of experimental Day 2 (135 minutes after drug intake). A German version of the Positive and Negative Affect Schedule (PANAS; Krohne, Egloff, Kohlmann, & Tausch, 1996) was used to track potential changes in subjective mood. Blood pressure and pulse were obtained using a Critikon Dinamap system (Tampa, FL) with a cuff placed on the right upper arm. Finally, saliva samples were collected using Salivette® collection devices (Sarstedt, Germany) and stored immediately at −18 °C (−0.4 °F) after testing. At the end of data collection, free cortisol concentrations were analyzed with a luminescence immunoassay (IBL International, Hamburg, Germany).

#### Pharmacological manipulation

In order to investigate the role of noradrenergic activation in potential stress effects on fear generalization, participants of the C+Prop and S+Prop groups received a small capsule which contained 40 mg of the β-adrenergic receptor antagonist propranolol 50 minutes before the stress or control manipulation. Participants of the C+Plac and S+Plac groups received identical looking placebo capsules. Dosage and timing of the drug were chosen in accordance with earlier studies that tested the role of noradrenergic arousal in learning and memory processes (Kroes et al., 2016; Schwabe, Nader, Wolf, Beaudry, & Pruessner, 2012; Schwabe et al., 2009). To verify the action of the drug, we analyzed the change in blood pressure and pulse measurements across experimental Day 2.

#### Control variables

To control for individual differences in subjective chronic stress, depressive mood, and anxiety, participants completed the Trier Inventory for the Assessment of Chronic Stress (TICS; Schulz & Schlotz, 1999), the German version of the Beck Depression Inventory (BDI-II; Beck, Steer, & Brown, 1996), the German version of the State-Trait Anxiety Inventory (STAI; Spielberger & Syndeman, 1994), and the social interaction anxiety scale (SIAS; Stangier, Heidenreich, Berardi, Golbs, & Hoyer, 1999). In addition, we assessed the quantity and quality of participants’ sleep over the past 4 weeks and the night between the two experimental days with a modified German version of the Pittsburgh Sleep Quality Index (PSQI; Buysse, Reynolds III, Monk, Berman, & Kupfer, 1989). To validate the successful blinding of the pharmacological manipulation, participants were asked to indicate what they thought which treatment they had received (treatment guess) at the end of the second experimental day.

#### General procedure

All testing took place between 1:00 pm and 7:30 pm on two consecutive days, with fear acquisition on Day 1 and experimental manipulations and the test of fear generalization on Day 2. The distribution of fear acquisition and test of generalization across 2 days allowed us to isolate stress effects on fear memory generalization, while ruling out influences on early consolidation processes.

##### Day 1 – Baseline phase and fear acquisition

Upon participants’ arrival at the lab, baseline measurements of vital signs (i.e., blood pressure and pulse), mood and saliva samples were taken. Afterwards an electrode for the electrical stimulation, serving as US, was placed on participants’ back of the right hand. For skin conductance recordings, two electrodes were attached to the left hand. Then, the individual pain threshold was determined using an adaptive testing procedure (QUEST procedure; Watson & Pelli, 1983) to obtain a shock intensity for every participant individually that was unpleasant but not painful, i.e., aiming at a 5 on a scale from 1 (no pain) to 10 (worst pain possible). Next, the baseline phase of the fear generalization paradigm started, followed immediately by the fear acquisition phase (Figure 1C). At the end of Day 1 testing, the pain strength rating as well as vital signs and mood were measured again and another saliva sample was taken.

##### Day 2 – Experimental manipulations and test phase

Same as on Day 1, at the beginning of Day 2, baseline measurements of vital signs, mood and a saliva sample were taken. Depending on the experimental condition, participants then received orally either a placebo or 40 mg of propranolol. During the latency period of 50 minutes, participants filled out the questionnaires before the TSST or control manipulation started. Then, participants completed an unrelated, nonarousing task for ~10 minutes, followed by the determination of the individual pain threshold. Next, approximately 30 minutes after stress onset, the critical test of fear generalization started, followed immediately by the perceptual discrimination task. Finally, participants were asked to indicate their treatment guess to check for successful blinding before participants were debriefed and compensated for participation.

#### Electrical stimulation and SCR analysis

**Figure 2.**
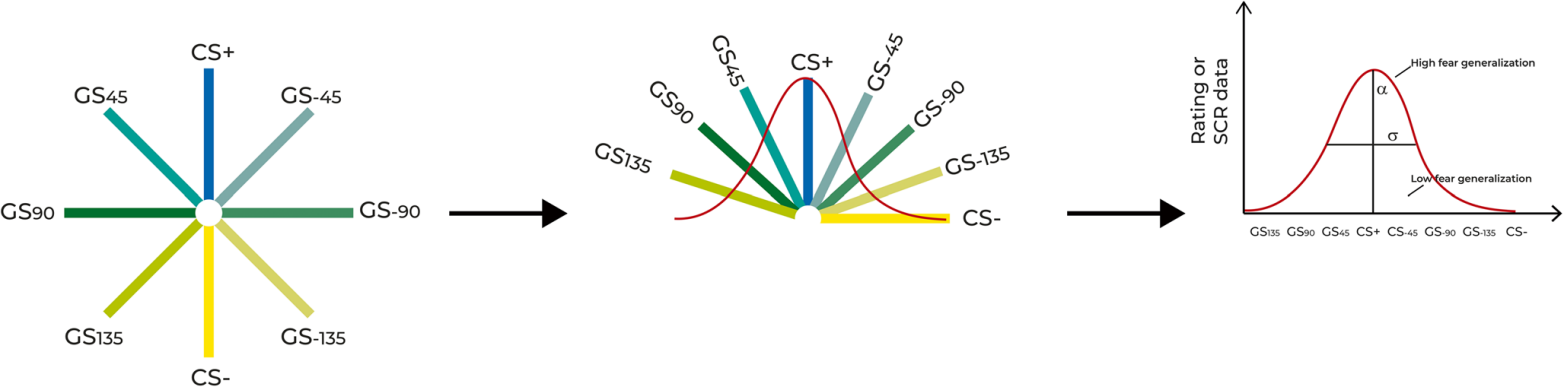
Gaussian-shaped fear-tuning. Bringing the circularly organized stimuli into a 2D coordinate space, it allowed us to fit a Gaussian function—defined through the parameters α (amplitude) and σ (width)—onto the individual responses to the stimuli.

The US consisted of trains of 5-ms electrical pulses at 66 Hz lasting in total 100 ms, applied via a constant voltage stimulator (STM200, BIOPAC Systems, Goleta USA) with a surface bar electrode attached to the back of the right hand. Electrodermal activity was recorded from the distal phalanx of the index and middle fingers of the left hand, using two 8mm Ag/AgCl electrodes, connected to a MP-150 BIOPAC System (BIOPAC Systems, Goleta USA), and assessed according to common guidelines (Boucsein et al., 2012). A deconvolution technique as implemented in Ledalab version 3.4.9 (Benedek & Kaernbach, 2010) was used to divide raw skin conductance recordings into the slowly varying tonic activity, i.e., skin conductance level, and more quickly varying phasic activity, i.e., skin conductance responses (SCRs). Skin conductance data were downsampled to a resolution of 20 Hz and optimized using four sets of initial values. To obtain the SCRs in response to the different CSs, we derived the average phasic driver within a response window from 1 s to 4 s after stimulus onset. The minimum amplitude threshold was set to 0.01 μS. Zero-responses were omitted from analyses. We calculated SCRs associated with the onset of individual faces at a single subject level, but excluded reinforced CS+ trials, to avoid confounds in SCR change due to electrical stimulation. To correct for interindividual differences, SCRs were z-transformed separately for the three different phases (Ben-Shakhar, 1985). Finally, responses to the different stimuli were averaged and single subject fear-tuning profiles for each phase were derived (Onat & Büchel, 2015; Figure 2).

#### Analysis of fear-tuning profiles

We expected participants to show the highest response to the CS+ and, with decreasing similarity to the CS+, decreasing responses to the other faces and thus to obtain Gaussian shaped fear-tuning profiles (Figure 2). Those individual fear-tuning profiles were analyzed using MATLAB (Release 2016b, Natick, MA). To characterize the fear-tuning, a Gaussian model with two parameters (α, amplitude, i.e., the strength of specificity; σ, tuning width (full width at half maximum), i.e., the strength of fear generalization) was used. We restricted our Gaussian model to be centered on the CS+-face. Fear-tuning profiles were calculated for zSCR and rating data separately. For further statistical analyses, we extracted the amplitude and width parameters of each profile (Onat & Büchel, 2015). For displaying our data of the fear generalization phase, we applied a Gaussian curve fitting function as implemented in SigmaPlot version 14.0 (Systat Software, Inc.).

#### Statistical data analysis

Statistical analyses were performed with SPSS 25.0 (IBM) and JASP 0.8.1.2 (JASP Team). Because there was no experimental manipulation on Day 1, the respective data were subjected to ANOVAs with the between-subject factor group with four levels (C+Plac, C+Prop, S+Plac, S+Prop). Data of Day 2 were subjected to ANOVAs with the two between-subjects factors condition (stress vs. control) and drug (placebo vs. propranolol). To validate the successful stress and drug manipulation, we used mixed-design ANOVAs with time as within-subject factor and the same two between-subject factors. To analyze the perceptual discrimination ability, a discrimination score was calculated by subtracting the mean false-alarm rate from the mean hit rate. To avoid extinction learning during the fear generalization test, participants still received the US in ~23% of the trials. To investigate, if this reinforcement had an impact on fear generalization on Day 2, we calculated proximity bins by counting the number of trials between the US and the different CS. We calculated the mean and grouped the proximity to US occurrence in three percentiles, whereby we obtained four bins: before any US had occurred; 1-11 trials after US occurrence; 12-26 trials after US occurrence; and >26 trials after US occurrence. We then performed a fear-tuning analysis for the stimuli dependent on the US-proximity and extracted the same parameters as for the general fear-tuning. Significant main or interaction effects were pursued using post-hoc planned comparisons, with Sidak correction if indicated. If the sphericity assumption was violated, Greenhouse-Geisser correction was applied.

To complement our inference statistical results, we additionally analyzed our main hypotheses with Bayesian statistics. This approach allows us to follow-up on possible nonsignificant results, to collect evidence for the null hypothesis, and thus to provide evidence for the presence or absence of an effect (Marsman & Wagenmakers, 2016). Because we had no specific information about our prior distribution, we chose the default Cauchy of 0.707. For paraphrasing the size of a Bayes Factor (BF), we followed the most common system, which suggest that a BF of 1-3 can be interpreted as anecdotal evidence, a BF of 3-10 as moderate evidence and a BF > 10 as strong evidence for the tested hypothesis (Jeffreys, 1961; Lee & Wagenmakers, 2013).

#### Study II

To determine the influence of stress on fear generalization, we include data from a second study that used the exact same behavioral paradigm as Study I in combination with a stress (TSST) or control manipulation but also included fMRI measurements, the results of which will be reported elsewhere. Seventy-three, healthy, right-handed volunteers (39 women) participated in this second study. In Study II, we also used a 2-day, between-subjects design, in which participants were pseudo-randomly assigned to the stress or control group. Applying the same predefined criterion for successful (explicit) fear acquisition on experimental Day 1 as in Study I, a final sample of 64 participants (34 women; age: *M* = 25.5 years, *SEM* = 0.51 years; Control: n = 31, Stress: n = 33) entered the data analyses. Same as in Study I, we assessed the same baseline measurements on Day 1, followed by the baseline phase and fear acquisition phase of the fear generalization paradigm. Approximately 24 h later, participants returned to the lab, provided again baseline measurements, and because there was no pharmacological manipulation in this study, the TSST/control manipulation followed immediately afterwards. After providing another saliva sample and vital signs measurement, participants were placed into an MRI scanner and completed the test phase of fear generalization, about 60 minutes after the onset of the stress/control manipulation. Afterwards, we obtained another saliva sample and measurement of vital signs, followed by the perceptual discrimination task outside of the scanner. Finally, participants were debriefed and compensated for participation.

Electrical stimulation and recordings of SCRs were comparable to Study I. Because of MRI acquisition on Day 2, however, electrical stimulation was applied to the lower right leg on both experimental days. Furthermore, we used MRI compatible equipment on Day 2 (BIOPAC Systems, Goleta USA). SCR data analysis and analysis of fear-tuning profiles were done in the exact same manner. Because we only had one between-factor in Study II (condition: stress vs. control), statistical analyses differed slightly, but the general procedure was the same. Data of Study I (placebo groups only) and Study II were merged for the Bayesian analysis.

## Results

### Study I

#### Day 1: Successful fear acquisition

At the beginning of experimental Day 1, baseline measurements of vital signs, mood, and salivary cortisol samples revealed no differences between groups (all *F* ≤ 1.999; all *p* ≥ 0.119; all *η*^*2*^ ≤ 0.056; Table 1). The analysis of the estimated pain threshold suggested a trend for a group effect (*F*(3,105) = 2.228, *p* = 0.089, *η*^*2*^ = 0.060), indicating a slightly higher pain threshold for the S+Plac group compared with the other three groups. However, this difference was not significant and more importantly, there was no group difference regarding the pain strength rating (*F*(3,105) = 0.825, *p* = 0.483, *η*^*2*^ = 0.023; Table 1), suggesting that the experimental groups evaluated the electrical stimulation as equally unpleasant.

**Table 1.** Physiological, endocrine, and subjective baseline measures on experimental Day 1

Variable	C+Plac	C+Prop	S+Plac	S+Prop
Salivary cortisol (nmol/L)	5.69 (0.64)	4.50 (0.66)	3.54 (0.65)	4.12 (0.67)
Systolic BP (mmHG)	134.25 (3.72)	140.69 (3.79)	136.76 (3.66)	136.92 (3.94)
Diastolic BP (mmHG)	76.61 (1.75)	78.48 (1.79)	78.40 (1.72)	78.22 (1.86)
Pulse (bpm)	84.77(2.88)	85.72 (2.94)	82.93 (2.83)	79.32 (3.10)
Positive affect	2.98 (0.11)	3.04 (0.11)	2.96 (0.11)	2.89 (0.12)
Negative affect	1.27 (0.08)	1.33 (0.08)	1.34 (0.08)	1.35 (0.08)
Pain threshold (V)	39.67 (2.27)	38.45 (2.31)	45.90 (2.23)	39.75 (2.40)
Pain strength	5.36 (0.35)	5.19 (0.36)	5.43 (0.35)	5.96 (0.37)

Data represent mean (standard error of the mean). Positive and negative affect represent scores of the positive and negative affect scale.

**Figure 3.**
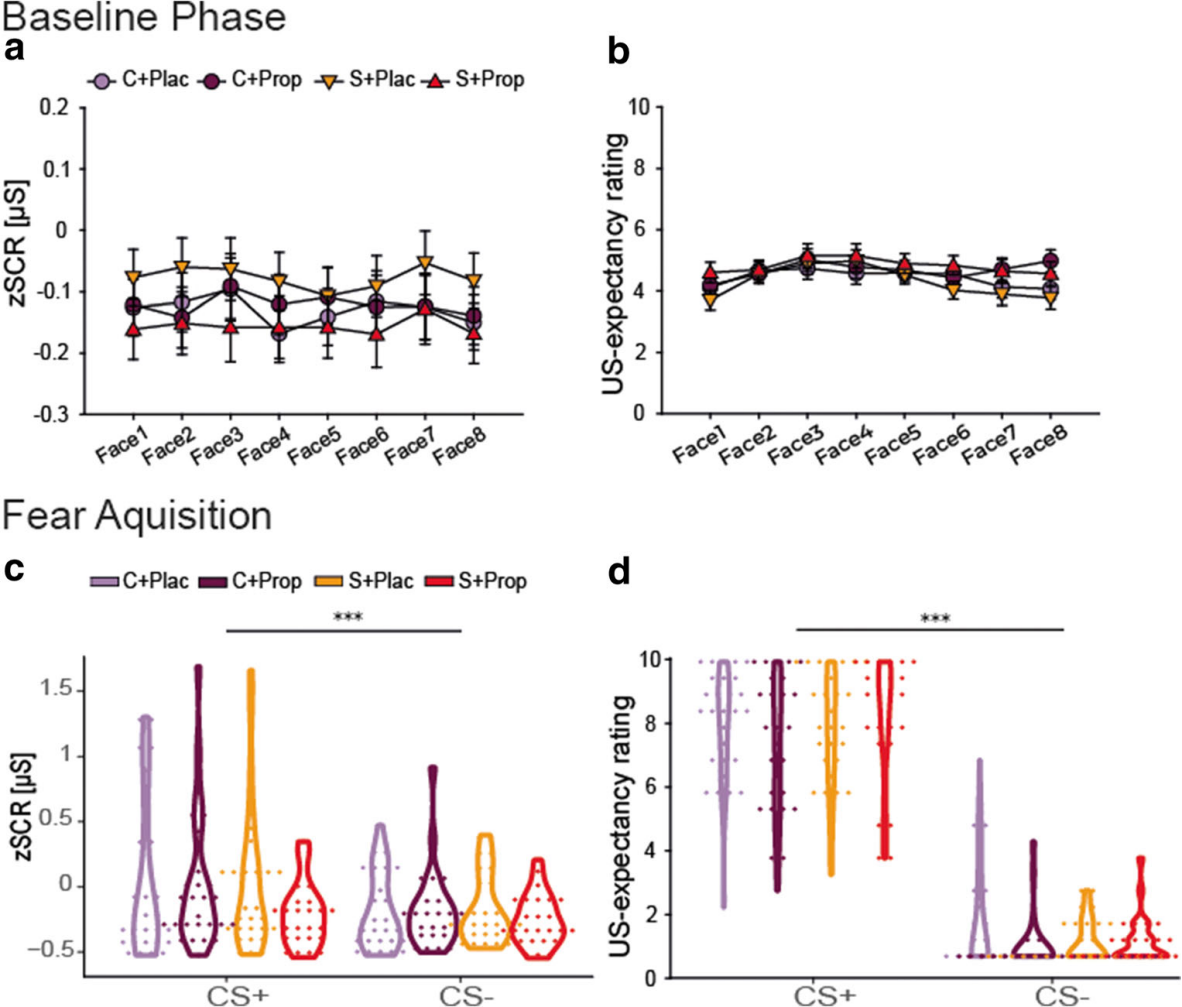
Day 1: Physiological and subjective responses to the face stimuli during the baseline and fear acquisition phases. (**A)** zSCR data as well as (**B**) explicit rating data showed no systematic a priori differences between faces and no group differences during or after the baseline phase. During and after fear acquisition, both (**C**) zSCR data as well as (**D**) explicit rating data showed successful fear learning reflected in higher responses to the CS+ than to the CS− in each group. Exclusion of the outliers do not affect our results. Error bars represent standard errors of the mean. Asterisks denote differences between stimuli (***p < 0.001).

As expected, because the US in the baseline phase was always signaled by a shock symbol and not associated with a certain stimulus, there were neither main effects nor a face stimulus × group interaction effect for the zSCR data (all *F* ≤ 1.75.8; all *p* ≥ 0.126; all *η*^*2*^ ≤ 0.017; Figure 3A). Analysis of the rating data however showed a main effect of face stimulus (*F*(2.97,305.94) = 3.838, *p* = 0.010, *η*^*2*^ = 0.036), without any influence of group (both *F* ≤ 1.186; both *p* ≥ 0.319; both *η*^*2*^ ≤ 0.033; Figure 3B). Post-hoc comparisons indicated that the first face stimulus was associated with a slightly lower US-expectancy (*M* = 4.13, *SD* = 1.64) than the third (*M* = 4.94, *SD* = 1.89; *p* = 0.014) and the fourth (*M* = 4.86, *SD* = 1.92; *p* = 0.043) face stimulus. However, all of these values reflect a rather high uncertainty about which stimulus is followed by a shock and there was no group main or face stimulus × group interaction effect (both *F* ≤ 1.1869; all *p* ≥ 0.319; all *η*^*2*^ ≤ 0.033).

Importantly, all groups showed successful fear acquisition, reflected in both a higher zSCR and a higher US-expectancy rating for the fear conditioned CS+ compared with the CS- (both *F ≥* 30.614; both *p* < 0.001; both *η*^*2*^ ≥ 0.227; Figure 2C and D, respectively), without any differences between groups (all *F* ≤ 2.412; all *p* ≥ 0.071; all *η*^*2*^ ≤ 0.017). Post-hoc *t*-tests indicated successful fear acquisition for all groups in the zSCR data (all *t* ≥ 2.13; all *p* ≤ 0.044; all *d* ≥ 0.425) and in the rating data (all *t* ≥ 12.543; all *p* < 0.001; all *d* ≥ 2.371).

### Day 2

#### Successful stress manipulation and validated drug action

**Figure 4.**
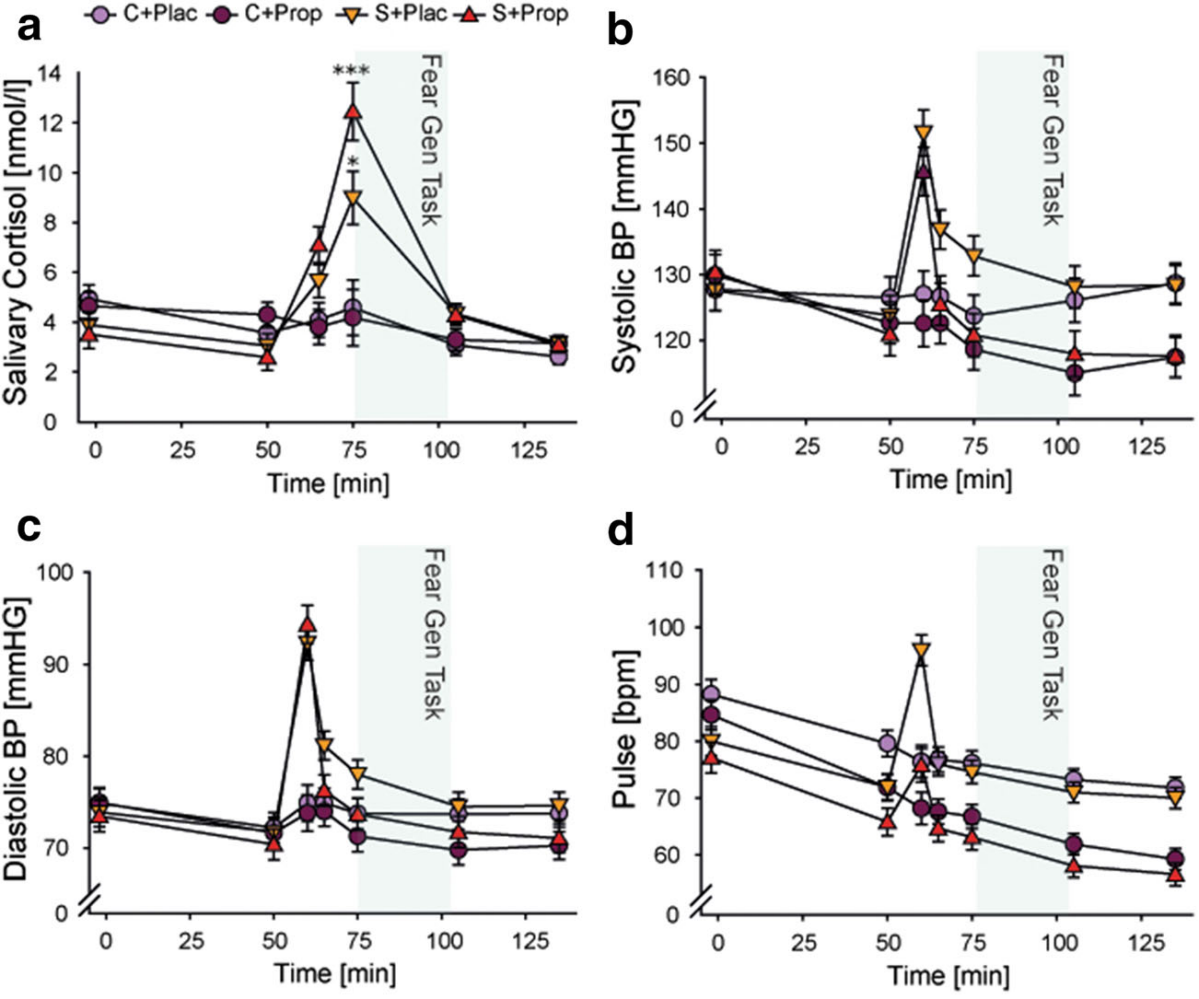
Pharmacological and stress manipulation check. (**A**) Salivary cortisol increased in response to the stress manipulation but was not affected by the pharmacological manipulation. (**B**) Systolic and (**C**) diastolic blood pressure, as well as (**D**) pulse increased during the stress compared with the control manipulation. However, the pharmacological manipulation resulted in reduced vital signs for the S+Prop group compared with the S+Plac group afterwards. Error bars represent standard errors or the mean. Asterisks denote difference between condition (stress vs. control). (*p < 0.05, **p < 0.01, ***p < 0.001).

Baseline measurements on Day 2 showed no differences between groups (all *F* ≤ 1.335; all *p* ≥ 0.267; all *η*^*2*^ ≤ 0.037; Figure 4), except for pulse (*F*(3,105) = 3.506, *p* = 0.018, *η*^*2*^ = 0.091). Post-hoc comparisons corrected for multiple testing revealed that the S+Prop group showed a significantly lower pulse than the C+Plac group (*p* = 0.022). We therefore included the baseline pulse as a covariate when analyzing treatment-related changes in pulse.

Significant changes in salivary cortisol, blood pressure, and pulse confirmed the effectiveness of our stress and drug manipulation (Figure 4). For salivary cortisol, in addition to the main effects of time and condition (both *F ≥* 31.783; both *p* < 0.001; both *η*^*2*^ ≥ 0.236), we obtained the expected significant time × condition interaction (*F*(1.758,181.059) = 31.783, *p* < 0.001, *η*^*2*^ = 0.236) and a significant time × condition × drug interaction (*F*(1.758,181.059) = 1.426, *p* = 0.019, *η*^*2*^ = 0.040). Post-hoc *t*-tests showed a significantly higher concentration of salivary cortisol for the stress group compared with the control group after the TSST/control manipulation that lasted until after the fear generalization test (all *p* ≤ 0.009). Post-hoc tests for the separate time points revealed a trend for a condition × drug interaction for the time point before the test of fear generalization (*F*(1,104) = 3.043, *p* = 0.084, *η*^*2*^ = 0.028), driven by a trend for higher cortisol concentrations in the S+Prop group compared with the S+Plac group (*p* = 0.092) and no difference between the two control groups (*p* = 0.709). Importantly, before the test of fear generalization started, both stress groups showed significantly higher salivary cortisol concentrations than both control groups (all *p* ≤ 0.029).

The analysis of vital signs revealed for systolic and diastolic blood pressure as well as for pulse significant time × condition and time × drug interaction effects (all *F ≥* 4.510; all *p* ≤ 0.003; all *η*^*2*^ *≥* 0.042; Figure 4), showing that the stress manipulation led to an increase in vital signs whereas propranolol decreased blood pressure and pulse. Only for pulse, there was a time × condition × drug interaction (*F*(2.223,228.967) = 4.634, *p* = 0.008, *η*^*2*^ = 0.043). Post-hoc t-tests, however, confirmed that both groups that had received propranolol showed significantly lower pulse than the placebo groups (all *p* ≤ 0.029).

As expected, condition—but not drug—influenced the subjective stress response. Both stress groups, irrespective of the pharmacological manipulation (all *p* ≥ 0*.*152), rated the treatment as significantly more difficult, stressful, and unpleasant than the control groups (all *p* ≤ 0.001; Table 2). In addition, we obtained a trend for a main effect of drug in the unpleasantness rating (*p* = 0.062). Post-hoc tests, however, revealed no significant difference between the placebo and propranolol groups (*p* = 0.119).

**Table 2.** Subjective responses on experimental Day 2

Variable	C+Plac	C+Prop	S+Plac	S+Prop
Positive affect				
Baseline	3.08 (0.13)	2.91 (0.13)	2.87 (0.12)	2.80 (0.13)
+ 50 min	2.88 (0.14)	2.63 (0.14)	2.52 (0.13)	2.61 (0.14)
+ 65 min	2.86 (0.13)	2.93 (0.13)	2.55 (0.13)*	2.60 (0.14)*
+ 75 min	2.61 (0.15)	2.54 (0.15)	2.45 (0.14)	2.49 (0.15)
+ 105 min	2.32 (0.14)	2.27 (0.14)	2.20 (0.13)	2.18 (0.14)
+ 135 min	2.31 (0.14)	2.34 (0.14)	2.28 (0.14)	2.17 (0.15)
Negative affect				
Baseline	1.21 (0.07)	1.36 (0.07)	1.30 (0.07)	1.30 (0.07)
+ 50 min	1.14 (0.05)	1.18 (0.05)	1.18 (0.05)	1.23 (0.05)
+ 65 min	1.18 (0.11)	1.20 (0.11)	2.01 (0.11)***	1.49 (0.12)***
+ 75 min	1.18 (0.09)	1.18 (0.09)	1.62 (0.09)*	1.21 (0.10)*
+ 105 min	1.27 (0.06)	1.15 (0.06)	1.36 (0.06)	1.15 (0.07)
+ 135 min	1.11 (0.04)	1.09 (0.04)	1.14 (0.04)	1.09 (0.04)
Pain threshold	43.56 (2.33)	40.65 (2.41)	49.87 (2.29)	43.22 (2.47)
Pain strength	6.19 (0.36)	5.52 (0.36)	5.89 (0.35)	6.56 (0.38)
TSST Questionnaire				
Difficulty	3.68 (0.42)	3.56 (0.43)	8.03 (0.41)***	7.48 (0.44)***
Unpleasantness	3.46 (0.43)	2.93 (0.43)	7.93 (0.42)***	6.84 (0.45)***
Stress	3.07 (0.41)	3158 (0.42)	7.69 (0.41)***	6.56 (0.44)***
Control variables				
STAI-S	35.32 (5.58)	38.48 (8.09)	38.45 (6.67)	38.60 (6.99)
STAI-T	36.07 (7.71)	36.41 (9.82)	37.69 (7.80)	38.60 (7.80)
BDI-II	5.86 (5.28)	5.47 (5.77)	8.55 (6.58)*	7.56 (3.97)*
TICS	11.54 (7.35)	12.89 (11.19)	16.00 (8.40)	14.36 (8.41)
SIAS	1.04 (0.61)	0.88 (0.66)	1.05 (0.51)	1.14 (0.56)
PSQI	7.14 (4.68)	8.07 (4.90)	8.66 (4.58)	8.44 (4.16)
Sleep quality between the days	75.25 (16.65)	70.70 (15.68)	66.86 (20.61)*	59.16 (26.22)*

Data represent mean (standard error of the mean). Positive and negative affect represent scores of the positive and negative affect scale. STAI = State-Trait Anxiety Inventory; BDI-II = Beck Depression Inventory; TICS = Trier Inventory for the Assessment of Chronic Stress; SIAS = Social Interaction Anxiety Scale; PSQI = Pittsburgh Sleep Quality Index. Asterisks denote differences between condition factor (stress vs. control) (*p < 0.05; ***p < 0.001).

As on Day 1, we tested whether the stress or drug manipulation affected the pain threshold and pain strength rating. Results revealed a main effect of drug in the estimated pain threshold (*F*(1,104) = 4.050, *p* = 0.047, *η*^*2*^ = 0.037) and a trend toward a main effect of condition (*F*(1,104) = 3.491, *p* = 0.065, *η*^*2*^ = 0.032). After correcting for multiple comparisons, post-hoc tests revealed that the S+Plac group had a higher pain threshold than the C+Plac group (*p* = 0.039). With respect to the pain strength rating, there was a trend for a condition × drug interaction (*F*(1,103) = 3.383, *p* = 0.069, *η*^*2*^ = 0.032), but post-hoc comparisons showed no significant difference between groups (all *p* > 0.255), i.e., groups experienced the electrical stimulation as comparably unpleasant.

#### Acute stress leaves fear generalization unaffected

**Figure 5.**
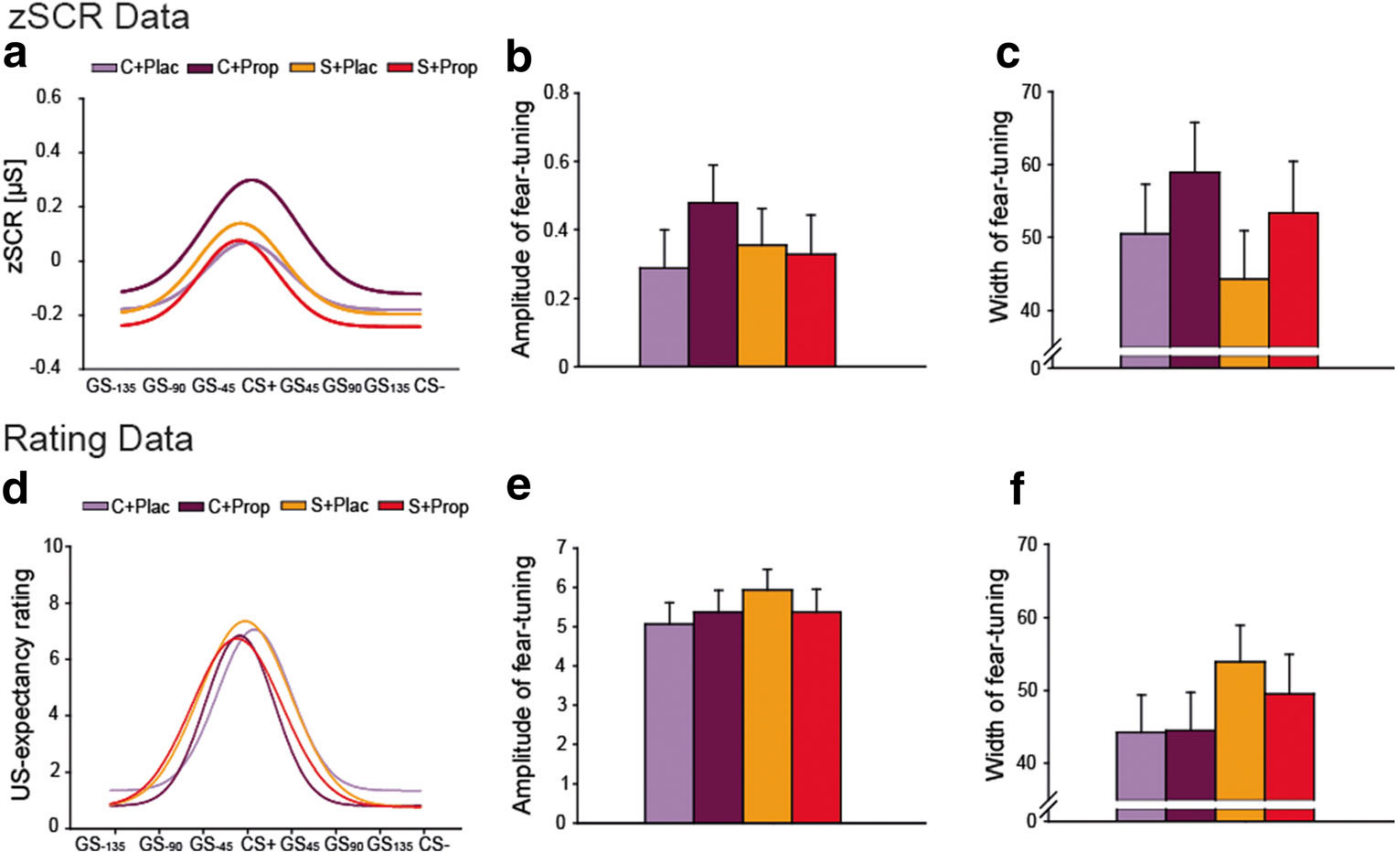
Day 2: Fear generalization phase. (**A**) Fear-tuning of zSCR to different stimuli during the test phase. No significant difference was seen between groups neither in (**B**) the strength of responding to the CS+ nor in (**C**) the fear generalization in the zSCR data. (**D**) Fear-tuning of US-expectancy rating to different stimuli after the test phase. Results of fear tuning of the rating data mirrored those obtained with the zSCR data. No significant difference was seen between groups neither in (**E**) the strength of responding to the CS+ nor in (**F**) the fear generalization.

Analyses of the fear-tuning parameters (amplitude and width) for the zSCR and the rating data revealed that fear memory specificity (amplitude), as well as fear generalization (width) were neither affected by condition nor by drug (all *F* ≤ 1.998; all *p* ≥ 0.160; all *η*^*2*^ ≤ 0.019; Figure 5). When specifically focusing on the differentiation ability between the fear conditioned CS+ and the safety signaling CS−, results showed a significant main effect of stimulus (both *F ≥* 37.415; both *p* < 0.001; both *η*^*2*^ ≥ 0.265), indicating that the acquired fear was still present. However, the absence of any main or interaction effect including the factors condition or drug (all *F* ≤ 2.894; all *p ≥* 0.092; all *η*^*2*^ ≤ 0.027) suggested that neither acute stress nor propranolol affected the differentiation ability.

**Figure 6.**
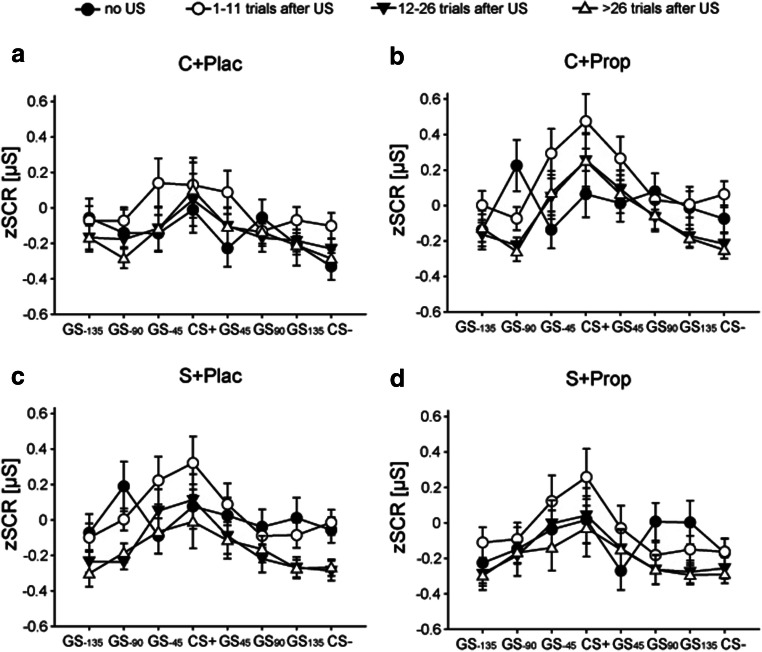
Fear-tuning dependent on US distance. Fear-tuning amplitude was dependent on US-proximity, i.e., all groups showed a higher fear-tuning amplitude, when they recently were reminded of the US-CS+ association. In contrast, the width of fear-tuning was unaffected by US-proximity across groups.

In a next step, we analyzed a possible influence of the US proximity; i.e., we aimed to test whether potential stress or drug effects might evolve only for stimuli occurring shortly after a reminder of the CS-US association (Figure 6). Analysis of zSCR data revealed a significant proximity effect (*F*(1.952,202.982) = 9.361, *p* < 0.001, *η*^*2*^ = 0.083), mirroring an increased amplitude of fear-tuning when the CS occurred shortly after the US. This was confirmed by post-hoc t-tests showing a significantly higher amplitude of all fear-tuning curves after a reminder US had occurred compared to trials presented before any US had occurred (all *p* ≤ 0.002). However, this effect was also neither influenced by condition nor by drug (all *F* ≤ 2.412; all *p* ≥ 0.094; all *η*^*2*^ ≤ 0.023). Analyzing the width of the fear-tuning revealed no significant main or interaction effects at all (all *F* ≤ 1.852; all *p* ≥ 0.140; all *η*^*2*^ ≤ 0.018), indicating that the width of fear-tuning remained unaffected by US-proximity for all our groups. Consequently, the lack of stress effects cannot be explained by an influence of US-proximity.

Finally, we analyzed the general perceptual discrimination ability. Results of this analysis revealed a main effect of condition (*F*(1,104) = 7.779, *p* = 0.006, *η*^*2*^ = 0.070), showing that both control groups (C+Plac: *M* = 0.61, *SD* = 0.07; C+Prop: *M* = 0.59, *SD* = 0.07) were better in discriminating the faces than the stress groups (S+Plac: *M* = 0.57, *SD* = 0.07; S+Prop: *M* = 0.55, *SD* = 0.08).

#### Control variables

The treatment guess at the end of the experiment indicated that participants were not aware of the administered drug. Most participants (74%) guessed that they had received a placebo, without any difference between the four groups (χ^2^(3) = 4.632, *p* = 0.201, Cramer’s *V* = 0.207). In addition, we obtained no group differences in terms of state, trait, or social anxiety (all *F* ≤ 1.524; all *p* ≥ 0.220; all *η*^*2*^ ≤ 0.014; Table 2). Furthermore, groups reported a comparable quantity and quality of sleep over the 4 weeks before testing (all *F* ≤ 1.133; all *p* ≥ 0.290; all *η*^*2*^ ≤ 0.011). However, we obtained a significant main effect of condition for the quality of sleep between the two test days (*F*(1,105) = 6.703, *p* = 0.011, *η*^*2*^ = 0.060), and for the level of depressive mood (*F*(1,105) = 4.532, *p* = 0.036, *η*^*2*^ = 0.042), suggesting a worse night of sleep and a higher level of depressive mood in the stress group. With respect to the level of chronic stress, results also revealed a trend for a main effect of condition (*F*(1,105) = 2.995, *p* = 0.086, *η*^*2*^ = 0.028), suggesting a higher level of chronic stress in participants of the stress group. To rule out that the aforementioned results are partly due to these group differences, we included these variables as covariates and re-run all our analyses. Controlling for these group differences, however, left our results largely unaffected, in particular there was no evidence for any stress-induced changes in fear memory generalization (width: all *p* ≥ 0.143; amplitude all *p* ≥ 0.178).

#### Study II: summary of results

**Figure 7.**
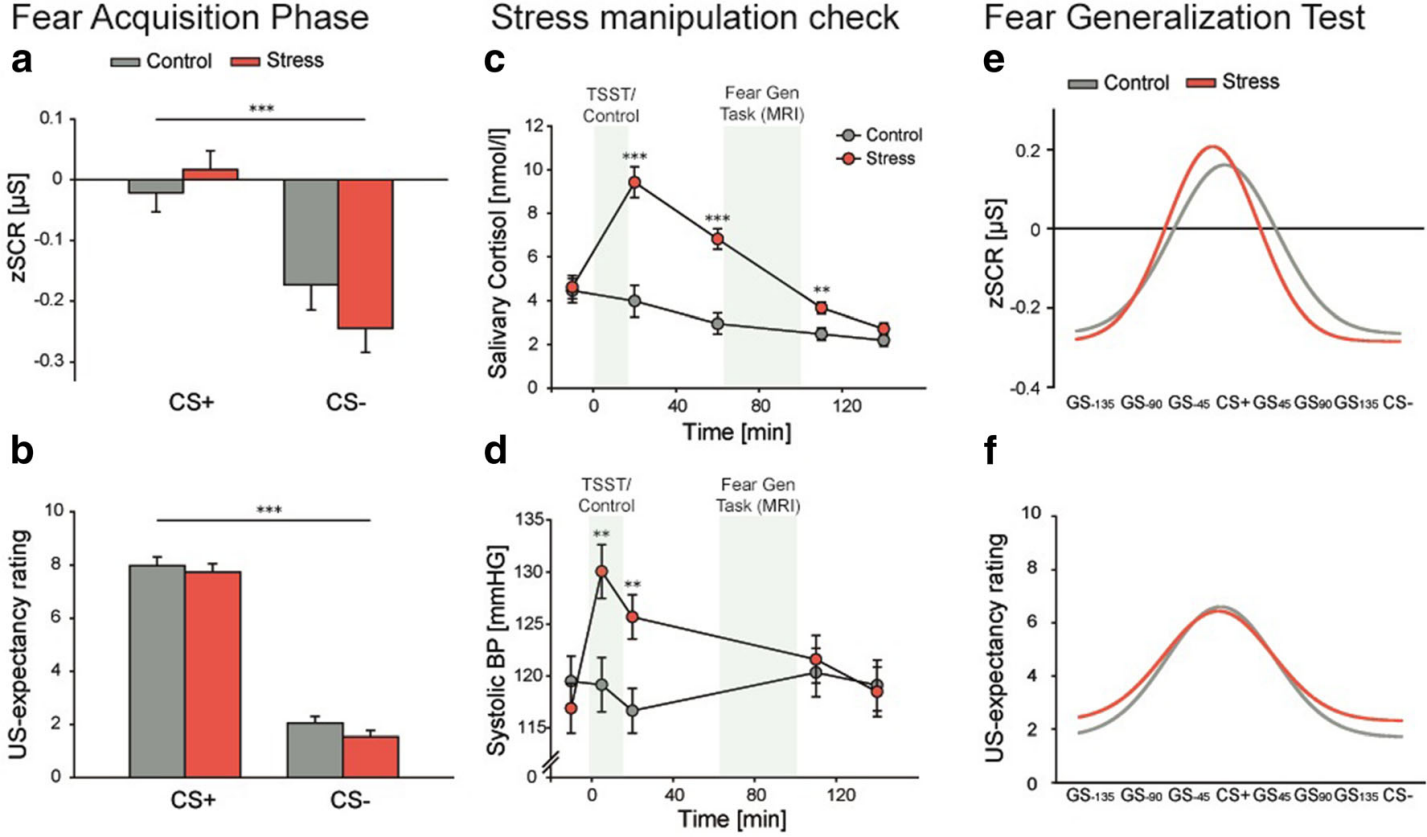
Results summary of Study II. Both groups showed successful fear acquisition on Day 1 in (**A**) zSCR data as well as (**B**) rating data. On Day 2, stress manipulation shortly before the test of fear generalization was successful, indicated exemplarily in an increase in (**C**) salivary cortisol and (**D**) systolic blood pressure in the stress group but not in the control group. The test of fear generalization revealed comparable fear-tuning curves for both groups across measurements, i.e., (**E**) zSCR data and (**F**) rating data.

We provide here a brief summary of the results of Study II (Figure 7). The detailed results of this study, including the fMRI data which are beyond the scope of the present manuscript, will be reported elsewhere.

At baseline on Day 1, participants of the stress and control groups did not differ in any of the baseline measurements, pain threshold or intensity (all *t* ≤ 1.292; all *p* ≥ 0.201; all *d* ≤ 0.326). During the acquisition phase, participants showed successful fear acquisition toward the CS+, indicated by a higher zSCR and subjective shock expectancy ratings for the CS+ compared with the CS− in the fear acquisition phase (both *F* ≥ 21.272; both *p* < 0.001; both *η*^*2*^ ≥ 0.255), without any differences between groups (all *F* ≤ 1.597; all *p* ≥ 0.211; all *η*^*2*^ ≤ 0.025; Figure 6A and B).

On Day 2, groups showed comparable baseline levels of subjective mood, salivary cortisol, blood pressure, pulse, and pain strength rating (all *t* ≤ 1.649; all *p* ≥ 0.104; all *d* ≤ 0.412). The subsequent stress induction via the TSST was successful as indicated by significant changes in subjective and physiological measurements. Participants of the stress group rated the TSST as significantly more challenging, uncomfortable, and stressful than the control group (all *t* ≥ −4.948; all *p* ≤ 0.001; all *d* ≥ 1.238) and showed an increase in salivary cortisol, blood pressure, and heart rate from before to after the manipulation in contrast to the control group (all *F* ≥ 6.251; all *p* < 0.001; all *η*^*2*^ ≥ 0.168; Figure 7C and D).

The fear-tuning curves obtained on Day 2 during the fear generalization phase showed no influence of the stress manipulation, neither for the amplitude nor for the width of fear-tuning for both of our measurements (all *t* ≤ 1.052; all *p* ≥ 0.297; all *d* ≥ 0.265; Figure 7E and F). Moreover, also the successful discrimination between CS+ and CS−, indicated by a significant stimulus main effect in both zSCR and rating data (both *F* ≥ 42.465; both *p* < 0.001; both *η*^*2*^ ≥ 0.410), did not differ between groups (all *F* ≤ 0.662 all *p* ≥ 0.419; all *η*^*2*^ ≤ 0.011). We further analyzed the influence of US-proximity and on the specific CS+/CS− discrimination. Same as in Study I, our analyses revealed a significant proximity effect for the amplitude of fear-tuning (*F*(1.796,107.779) = 12.138, *p* < 0.001, *η*^*2*^ = 0.168), with post-hoc comparisons showing a significantly lower fear response from before any US occurred to all proximity bins after US administration (all *p* ≤ 0.042). However, this effect was not influenced by group as there was neither a main effect of condition nor a group × proximity interaction effect (both *F* ≤ 0.323; both *p* ≥ 0.702; both *η*^*2*^ ≤ 0.005). Regarding the width of fear-tuning, Study II showed the same pattern of results as Study I, indicating no influence of US-proximity or group (all *F* ≤ 0.768; all *p* ≥ 0.513; all *η*^*2*^ ≤ 0.013).

Finally, in contrast to the results of Study I, analyses of the general perceptual discrimination ability revealed no significant group differences (*t*(62) = 0.321, *p* = 0.750, *η*^*2*^ = 0.080).

#### Bayesian analysis across studies I and II provides evidence for an absence of a stress effect on fear generalization

**Figure 8.**
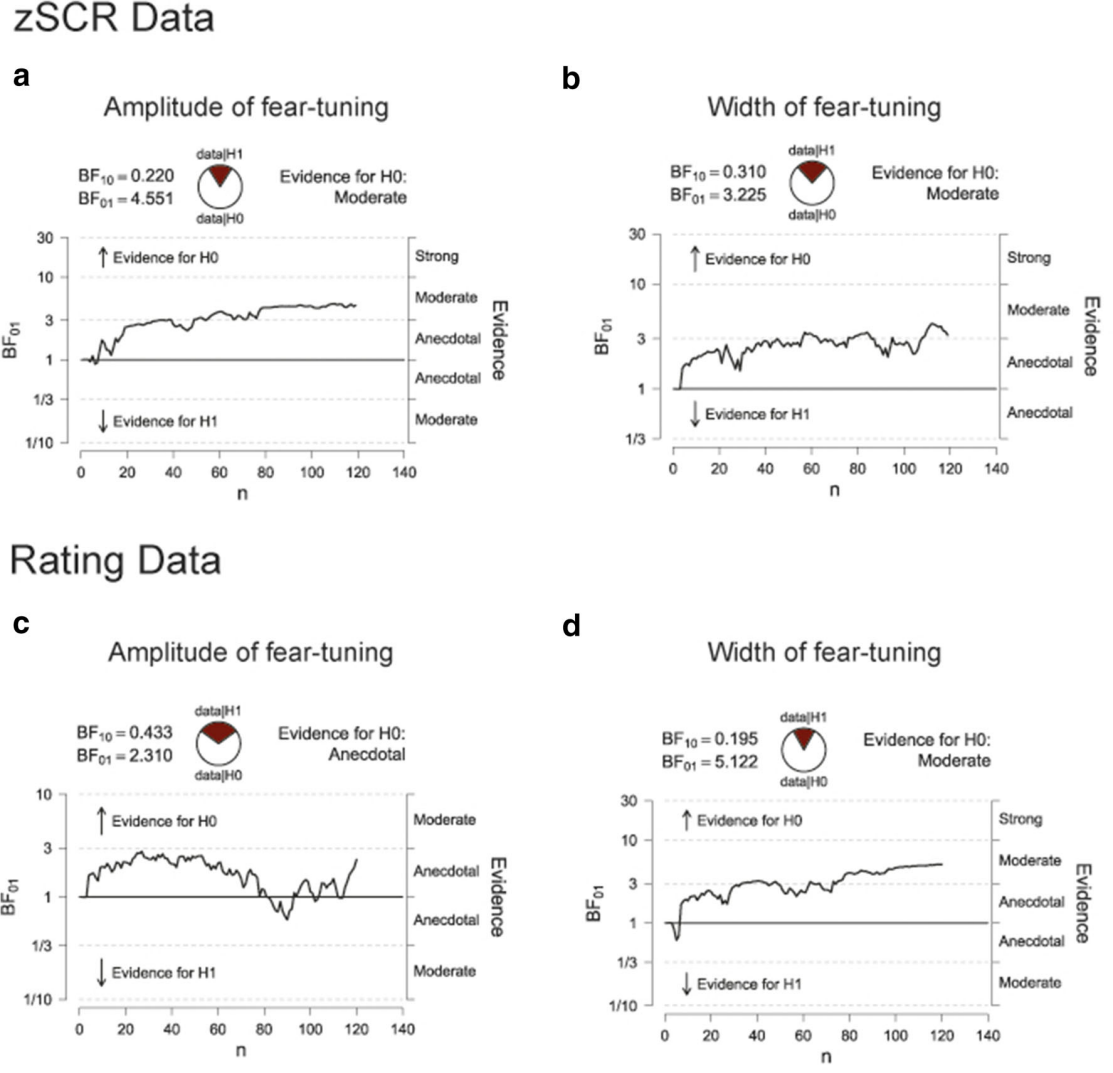
Flow of evidence for *H*_*0*_*.* With accumulating data, fear-tuning results of zSCR and rating data show rather evidence in favor of the *H*_*0*_*,* i.e., no influence of stress on fear-tuning, in contrast to the *H*_*1*_*,* i.e., there is an influence of stress on fear generalization. BF_01_ = Bayes Factor for the *H*_*0*_*.* BF_10_ = Bayes Factor for the *H*_*01*_.

Inference statistical results of Studies I and II converge in that they suggest that acute psychosocial stress has no influence on fear generalization in healthy participants. In order to assess the evidence in favor of the null hypothesis, we complemented these results with Bayesian analyses. Therefore, we combined the sample of Study II with the placebo groups of Study I (i.e., C+Plac and S+Plac; final sample n = 121) and analyzed the amplitude and width of our fear-tuning profiles obtained with our zSCR data and rating data with Bayesian independent samples *t*-tests. Results showed that the obtained Bayes factors for our analyses of fear-tuning amplitude and width for the zSCR and rating data provide evidence for the *H*_*0*_ (Table 3). Specifically, the Bayes factors indicate that it is 4.5 (zSCR data) and 5.1 (rating data) times more likely that the amplitude of our fear-tuning profiles does not differ between the stress and the control group. In addition, it is 3.2 (zSCR) and 2.3 (rating data) more likely that also the width of the fear-tuning profiles remains unaffected by the acute stress exposure. Figure 8 depicts the sequential analysis of the data, i.e. the evidential flow for the accumulating data. This visualization suggests that the data favors rather consistently and constantly the *H*_*0*_. However, it should be noted that this evidence for the *H*_*0*_ ranges between moderate (width of the rating data and amplitude and width of the zSCR data) and anecdotal (amplitude of the rating data). At the same time, the error percentage of all our analyses is ≤0.013%, which suggests a high stability of the underlying numerical algorithm that was used to obtain these results.

**Table 3.** Results of Bayesian independent samples *t*-test

Variable	BF_01_	Error %
zSCR data		
Amplitude of fear-tuning	4.551	0.004
Width of fear-tuning	3.225	0.001
Rating data		
Amplitude of fear-tuning	2.310	0.004
Width of fear-tuning	5.122	0.013

## Discussion

Our results showed no influence of stress on fear generalization, neither in autonomic responding (SCR), nor in verbal report (US-expectancy ratings). Based on these results, we included data of a second study that differed in some aspects (e.g., MRI vs. behavioral study) but used the exact same paradigm and reanalyzed our data with Bayesian statistics to test the evidence in favor of an absence of a stress effect on fear generalization. This analysis provided evidence that stress has no impact on fear generalization in a population of young healthy individuals. Likewise, the blockade of noradrenergic arousal through propranolol left fear generalization unaffected.

In contrast to the present results, previous evidence in rodents suggested that stress may increase fear generalization (Bender et al., 2018; Kaouane et al., 2012). However, findings in rodents are also heterogeneous. Whereas one study showed fear generalization after corticosterone injection (Kaouane et al., 2012), another study did not show such an influence (Bueno, de Paiva, Correa, Tiba, & Fornari, 2017). Obviously, species differences, for instance in metabolism or brain structure, might hamper the translation of findings from rodents to humans. However, in addition to species differences, there were important methodological differences between previous rodent studies and the present study, which may account for the partly discrepant results. First, there are differences in the timing of the stress induction. Previous animal studies exposed rats to stress either before (Bender et al., 2018) or immediately after (Kaouane et al., 2012) fear conditioning, which most likely affected initial fear acquisition and/or consolidation and thus makes it impossible to disentangle these effects from potential changes in the actual generalization of fear. In the present study, we exposed participants to stress 24 h after fear conditioning. After a traumatic event, people may suffer from flashbacks, nightmares, or intrusive memories, which again result in a marked stress response and may add to an increase in fear generalization. Our delayed stress manipulation therefore enabled us to isolate these later stress effects on fear generalization from those during initial fear acquisition or consolidation. In addition, the animal studies targeted primarily the influence of stress on contextual fear generalization, and one study explicitly showed no effect of glucocorticoid injection on cued fear generalization (Kaouane et al., 2012), which is in line with the present results. Finally, it should be noted that previously reported increases in fear generalization were obtained only when threat intensities were rather high and corticosterone levels exceeded a certain threshold (Kaouane et al., 2012). This is in line with another study in humans, which showed increased fear generalization only when the US intensity was rather high compared with low (Dunsmoor, Kroes, et al., 2017).

In our experiment, we explicitly instructed participants to determine a pain threshold of a moderate intensity, i.e., the electrical shock should be unpleasant but not painful. Yet, the only previous study in humans that investigated the influence of stress on fear generalization used nonpainful shocks as well but did obtain a stress effect (Dunsmoor, Otto, et al., 2017). There are, however, other variables that differ between this previous study and the present studies, which may explain the different findings. While the studies differ in the modality of the CS (auditory vs. visual) and the used stressor, the most significant difference relates to the learning schedule. Compared with the earlier study, the present studies had a lower reinforcement rate (40% vs. 23%) and used considerably more trials (20 and 64 trials vs. 123 and 293 trials), both during acquisition and during the generalization test. Accordingly, fear learning may have taken longer but may have been more intense in the present study, rendering it potentially less vulnerable to a subsequent stress manipulation. This would have been in line with the finding that partial reinforcement rates, in contrast to continuous reinforcement, weaken the development of conditioning (Dunsmoor, Bandettini, & Knight, 2007). At the same time, partial reinforcement rates are assumed to prolong fear memory extinction (Lonsdorf et al., 2017).

Because we did not obtain any stress effects on fear learning, it is not surprising that in addition, there were no interaction effects of stress and propranolol. Furthermore, our results neither revealed any effects of propranolol per se. This is in contrast to previous studies that showed an influence of propranolol on fear learning processes, such as extinction learning (Burhans, Smith-Bell, & Schreurs, 2018; Chalkia, Weermeijer, Van Oudenhove, & Beckers, 2019; but see Rodriguez-Romaguera, Sotres-Bayon, Mueller, & Quirk, 2009), fear memory reconsolidation (Kindt et al., 2009; Soeter & Kindt, 2011, 2012), or the return of fear memory (Kroes et al., 2016). However, these previous studies yielded partly inconsistent results. These inconsistencies may be due to the distinct fear learning processes under investigation, including extinction, reconsolidation, return of fear, and—in the present study—fear generalization. Moreover, it has been suggested that the administration of propranolol might primarily affect the fear-arousing aspects, reflected for instance in the startle response, but less in declarative aspects of fear memory, reflected in skin conductance responses, subjective distress, and expectancy ratings (Kindt et al., 2009; Soeter & Kindt, 2011, 2012). Furthermore, it has been shown that fear conditioning measured with the startle response is not dependent on conscious discriminative fear learning, whereas fear conditioning measure in SCR is (Sevenster, Beckers, & Kindt, 2014). In contrast to the SCR, the fear potentiated startle (FPS) does not decrease with repeated presentation of the same stimulus (Boucsein et al., 2012), and additionally, it can be evoked at other time points, independently of CS presentation, which makes it possible to compare a response to a specific CS with a baseline (Lonsdorf et al., 2017). However, studies combining SCR or FPS measurement with fMRI found a similar relationship regarding the neural underpinnings, such that the amygdala correlated with conditioned SCRs as well as conditioned FPS (MacNamara, Rabinak, Fitzgerald, Zhou, Shankman, Milad, & Phan, 2015; van Well, Visser, Scholte, & Kindt, 2012). Thus, differences in the obtained measures of fear might account for the discrepant findings between studies, and it cannot be completely ruled out that there might have been an influence of stress and/or propranolol in the present study if we had included additional measures, such as the startle response.

On a neural level, it has been shown that stress mediators act mainly on the hippocampus, amygdala, and prefrontal cortex (for a review see McEwen, Nasca, & Gray, 2016), all of which are known to play an important role in the process of fear generalization (Dunsmoor & Paz, 2015; Lissek, Bradford, et al., 2014; Onat & Büchel, 2015). One previous study in animals directly injected glucocorticoids into the hippocampus and found an increase in fear generalization only in contextual fear learning but not in cued fear learning (Kaouane et al., 2012). Results of another study that specifically investigated cued fear generalization (Pollack, Bezek, Lee, Scarlata, Weingast, & Bergstrom, 2018) are in line with our results, as they found an increase in fear generalization with passing time. In addition, their results suggest that cued fear generalization is, in part, dissociable from contextual fear generalization. Based on these results, one could assume that stress may have a higher impact on contextual fear generalization compared with cued fear generalization, which might explain the lack of a stress effect in our studies.

Finally, our results were not only consistent across (“declarative”) measures, i.e., shown in our SCR data as well as in our US-expectancy rating data, but also across independent experiments. A Bayesian analysis across these independent studies supported the conclusion that acute stress does not affect fear generalization in a population of healthy, young individuals. However, multiple studies in patients suffering from anxiety or stress-related disorders, such as generalized anxiety disorder (Lissek, Kaczkurkin, et al., 2014), social anxiety disorder (Ahrens et al., 2016), or panic disorder (Lissek et al., 2010) or PTSD (Kaczkurkin et al., 2017), showed a broader fear generalization gradient compared with healthy controls, supporting the idea of fear overgeneralization as a transdiagnostic marker across multiple fear-related disorders (Dymond, Dunsmoor, Vervliet, Roche, & Hermans, 2015). At the same time, there is broad evidence that stress impacts these fear-related disorders (de Quervain et al., 2017). Therefore, while we obtained no effect of acute stress on fear generalization in healthy individuals, there may well be an important effect in vulnerable populations, such as individuals at high-risk for anxiety disorders or PTSD. If stress increases fear generalization in these populations, testing whether a blockade of noradrenergic arousal might counteract this stress-induced fear overgeneralization would be highly relevant.
